# Description of a novel *Lankesterella* species (Apicomplexa: Eimeriorina) infecting the green iguana (*Iguana iguana*) from Eastern Amazonia

**DOI:** 10.1017/S003118202510139X

**Published:** 2026-03

**Authors:** Danilo Pelaes de Almeida, Amanda Maria Picelli, Carolina Romeiro Fernandes Chagas, Lucio André Viana

**Affiliations:** 1 Programa de Pós-Graduação em Biodiversidade Tropical, Universidade Federal do Amapá (UNIFAP), Macapá, AP, Brazil; 2 Laboratório de Estudos Morfofisiológicos e Parasitários, Departamento de Ciências Biológicas e da Saúde, UNIFAP, Macapá, AP, Brazil; 3 Department of Biology, Villanova University, Villanona, PA, USA; 4 Departamento de Parasitologia, Universidade Federal de Minas Gerais, Belo Horizonte, MG, Brazil; 5 State Scientific Research Institute Nature Research Centre, Vilnius, Lithuania

**Keywords:** *18S* rDNA, haemococcidia, haemoparasites, squamate, taxonomy

## Abstract

Through an integrative approach that combined microscopy and molecular analyses of the *18S* rDNA gene, this study describes a novel haemococcidian species, *Lankesterella nucleoflexa* sp. nov., and presents data on another *Lankesterella* sp. Both parasites were found in the green iguana (*Iguana iguana*) from Eastern Amazonia, Brazil. *Lankesterella nucleoflexa* sp. nov. is characterized by a unique nuclear plasticity; its nucleus exhibits variable shapes and condensation states, appearing condensed and seemingly divided when adjacent to the host cell nucleus and elongated when positioned opposite. This species infects erythrocytes, monocytes and heterophils, inducing significant nuclear deformities. Phylogenetic analysis placed both *Lankesterella* sequences in a clade with other *Lankesterella* parasites from lizards, highlighting the genetic diversity of this genus within this host group. These findings expand the knowledge about parasitic biodiversity in Neotropical reptiles and underscore the necessity of integrating morphological and molecular methodologies to elucidate the taxonomy of understudied groups such as haemococcidians.

## Introduction

The phylum Apicomplexa forms a diverse group of protozoan parasites with several representatives found infecting amphibians and reptiles, such as haemococcidians (Coccidia, Eimeriorina) of the genera *Lankesterella, Schellackia* and *Lainsonia* (Desser, 1993; Telford, 2009). These parasites are characterized as extra-intestinal coccidian parasites that invade the host’s blood cell and which development (merogony, gametogony and sporogony) occurs in the tissues (e.g. liver and intestine) of the same vertebrate host (Desser, 1993). Microgamonts produce large numbers of microgametes, and the naked oocysts produce variable numbers of sporozoites (8 in *Lainsonia* and *Schellackia*; and 32 or more in *Lankesterella*), which enter the blood cells (Landau, 1973; Desser, 1993). These sporozoites are ingested by invertebrate hosts (leeches, mites or mosquitoes) and transmitted to another vertebrate host during blood feeding of the invertebrate host, or by ingestion of the infected vector (Desser, 1993). No development is observed in invertebrate hosts (Telford, 2009).

Traditionally, haemococcidians have been described as part of the family Lankesterillidae, however molecular studies on *18S* ribosomal DNA (rDNA) gene sequence have supported the polyphyletic origin of the family, with *Lankesterella* and *Schellackia* as distantly related (Megía-Palma et al. 2014; 2017). Regarding *Lainsonia*, despite being considered synonymous with *Schellackia* based on life cycle characteristics (Levine, 1988; Megía-Palma et al. 2017), its Neotropical distribution coupled with the lack of available genetic data (Landau, 1973; Telford, 2009), and the possible restriction of *Schellackia* to Old World hosts (Megía-Palma et al. 2017) warrant further investigation into the validity of this genus. In addition, the absence and consistency of morphological diagnostic characteristics to differentiate parasites between genera means that their classification primarily relies on the characteristics of the oocyst during endogenous development (Telford, 2009; Megía-Palma et al. 2017). These taxonomic uncertainties reflect the lack of knowledge about haemococcidian diversity and phylogenetic relationships, which significantly hinders the accurate classification and understanding of the evolutionary relationships within this group of parasites.

To date, 17 haemococcidian species – 10 *Schellackia* spp., 5 *Lankesterella* spp., and 2 *Lainsonia* spp. – have been described in at least 37 lizard species across 10 families (*Agamidae, Anolidae, Chamaeleonidae, Iguanidae, Lacertidae, Phyllodactylidae, Phrynosomatidae, Polychrotidae, Scincidae* and *Teiidae*) in Africa, the Americas, Asia and Europe (Table 1). Although more than 30 haemococcidian sequences from reptiles are available in GenBank (Veith et al. 2023; Hajiyan and Javanbakht, 2022), only 4 named species have corresponding *18S* rDNA gene sequences (Megía-Palma et al. 2014; 2016, 2017; Chang et al. 2023): *Lankesterella desseri* (Chang et al. 2023), *Lankesterella golvani* (Rogier and Landau, 1975), *Lankesterella occidentalis* (Bonorris and Ball, 1955) and *Schellackia bolivari* (Reichenow, 1920). In the Brazilian Amazon, only 3 species have been described morphologically, all in the 1970s (Landau, 1973; Landau et al. 1974; Lainson et al. 1976): *Schellackia landaue* (Lainson et al. 1976); *Lainsonia iguanae* (Landau, 1973) and *Lainsonia legeri* Landau et al. 1974; (Table 1).

**Table 1.** List of haemococcidian species records in lizards, with GenBank accession numbers (partial 18S rDNA gene sequences available), locality and references Table 1 long description.

Haemococcidian species	Lizard species	GenBank	Locality	Reference
*Lainsonia iguanae*	*Iguana iguana*	N/A	Brazil; French Guiana; Panama; Venezuela	Landau (1973); Telford (1977); Telford (2009)
*Lainsonia legeri*^*a*^	*Tupinambis teguixin*	N/A	Brazil; Venezuela	Landau et al. (1974); Telford (2009)
*Lankesterella baznosanui*	*Zootoca vivipara*	N/A	Romania	Chiriac and Steopoe (1977)
*Lankesterella desseri*	*Ctenosaura similis*	OR425015–OR425032	Nicaragua	Chang et al. (2023)
*Lankesterella golvani*	*Anolis armouri*	N/A	Hispaniola	Telford (2009)
	*Anolis carolinensis*	KU180248	USA	Jordan and Friend (1971); Telford (1978); Megía-Palma et al. (2016)
	*Anolis chlorocyanus*	N/A	Hispaniola	Telford (2009)
	*Anolis conspersus*	N/A	Grand Cayman	Telford (2009)
	*Anolis cybotes*	N/A	Hispaniola	Telford (2009)
	*Anolis distichus*	N/A	Hispaniola	Telford (2009)
	*Anolis grahami*	N/A	Jamaica	Telford (2009)
	*Anolis lineatopus*	N/A	Jamaica	Telford (2009)
	*Anolis marmoratus*	N/A	Island of Guadeloupe	Rogier and Landau (1975)
	*Anolis opalinus*	N/A	Jamaica	Telford (2009)
	*Anolis pecuarius*	N/A	Hispaniola	Telford (2009)
	*Anolis ricordii*	N/A	Hispaniola	Telford (2009)
	*Anolis sagrei*	N/A	USA; Jamaica	Telford, 1978; Telford, 2009
*Lankesterella millani*	*Timon nevadensis*	N/A	Spain	Álvarez-Calvo (1975)
*Lankesterella occidentalis*	*Sceloporus occidentalis*	MF167550–MF167551	USA	Laveran and Pettit (1909); Todd and Wolbach (1912); Rogier (1977)
	*Sceloporus undulatus*	N/A	USA	Jordan and Friend (1971)
	*Uta stansburiana*	MF167552–MF167553	USA	Bonorris and Ball (1955); Megía-Palma et al. (2017)
*Schellackia agamae*	*Agama agama*	N/A	Senegal; Gambia; Nigeria; Central African Republic; Sudan	Laveran and Pettit (1909); Todd and Wolbach (1912); Rogier (1977)
	*Laudakia stellio*	N/A	Egypt; Israel	Bristovetzky and Paperna (1990); Omran et al. (1981); Ostrovska and Paperna (1987a, 1987b); Paperna (1992)
*Schellackia bocagei*	*Podarcis vaucheri*	N/A	Spain	Álvarez-Calvo (1975)
*Schellackia bolivari*	*Acanthodactylus erythrurus*	KJ131415–KJ131416	Spain; Morocco	Reichenow (1920); MegíaíPalma et al. (2014)
	*Psammodromus hispanicus*	N/A	Spain	Reichenow (1920)
*Schellackia brygooi*	*Calumma brevicorne*	N/A	Madagascar	Brygoo (1965)
	*Oplurus cuvieri*	N/A	Madagascar	Landau (1973)
	*Oplurus cyclurus*	N/A	Madagascar	Landau (1973)
*Schellackia calotesi*	*Calotes mystaceus*	N/A	Thailand; Myanmar	Finkelman and Paperna (1998); Telford (2009)
	*Calotes versicolor*	N/A	India; Thailand; Myanmar	Ray and Sarkar (1969); Finkelman and Paperna (1998); Telford (2009)
*Schellackia landaue*	*Polychrus marmoratus*	N/A	Brazil; Venezuela	Lainson et al. (1976); Telford (1980)
	*Polychrus gutturosus.*	N/A	Panama	Telford (1977)
*Schellackia mabuyai*	*Trachylepis striata*	N/A	Kenya	Mutinga and Dipeolu (1989)
*Schellackia ptyodactyli*	*Ptyodactylus hasselquistii*	N/A	Palestine	Paperna and Finkelman (1996)
*Schellackia orientalis*	*Takydromus sexlineatus*	KJ131414	Thailand; Indonesia	Telford (1993); MegíaíPalma et al. (2014)
	*Takydromus tachydromoides*	N/A	Japan	Telford (1993)
*Schellackia weinbergi^a^*	*Tupinambis teguixin*	N/A	French Guiana	Leger and Mouzels (1917)

a *Lainsonia legeri* was considered a synonym of *S. weinbergi* by Levine (1988).

The green iguana, *Iguana iguana* (Linnaeus, 1758), is a widespread lizard species in the American continent, ranging from southern North America to central-northern South America and present in some Caribbean islands (Ribeiro-Júnior, 2015). In Brazil, it is relatively common in the Amazonia, Caatinga and northern Cerrado biomes, often associated with wooded areas close to streams (Ribeiro-Júnior, 2015). It is primarily arboreal, with diurnal and herbivorous habits (Alberts et al. 2004). This iguanid lizard has been pointed as the host of some haemoparasite species, such as haemosporidians – *Plasmodium basilisci* (Peláez and Péres-Reyes, 1952) reported in El Salvador (Herban and Coatney, 1969), *Plasmodium minasense carinii* (Leger and Mouzels, 1917) in Brazil, Colombia, French Guiana and Trinidad (Ayala SC, 1978; Telford, 1979), *Plasmodium iguanae* (Telford, 1980) in Venezuela (Telford, 1980), *Plasmodium rhadinurum* (Thompson and Huff, 1944) in Brazil and Mexico (Thompson and Huff, 1944; Walliker, 1966; Telford, 1977, 1980); haemogregarines – *Hepatozoon iguanae* (Laveran and Nattan-Larrier, 1912) and *Hepatozoon sinimbui* (Carini, 1942) in Brazil (Laveran and Nattan-Larrier, 1912; Carini, 1942); and haemococcidian – *L. iguanae* in Brazil (Landau, 1973). However, to date, no molecular characterization has been conducted with the haemoparasites of this host.

Thus, by combining microscopy and molecular tools, we investigated the diversity of haemococcidians infecting green iguanas from the Eastern Amazonia, Brazil. This resulted in the description of 1 new species, *Lankesterella nucleoflexa* sp. nov., and molecular data on another *Lankesterella* sp. parasite.

## Materials and methods

### Sampling and microscopy analyses

**Figure 1.**
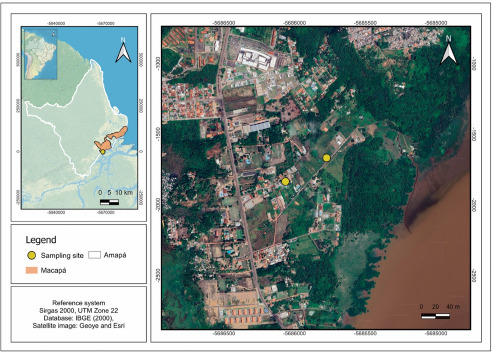
Map showing the collection sites (yellow circles) of green iguanas (*Iguana iguana*) in Macapá municipality, State of Amapá, Eastern Amazonia, Brazil. Sampling points were in forest fragments near urban areas. Figure 1 long description.

A total of 5 specimens of green iguanas (*I. iguana*) were manually captured between October 2022 and November 2023 in the metropolitan region of Macapá, State of Amapá, Brazil (Fig. 1). The capture sites included a forest fragment (0°00’53.9” S, 51°04’34.2” W), where 3 specimens were collected, and an urban area (0°00’59.3” S, 51°04’43.6” W), which yielded two specimens. To avoid resampling, individuals were photographed and identified based on sexual dimorphism and unique natural marks, such as scale patterns, scars and colouration. Blood samples were taken via caudal venipuncture (Samour and Hart, 2020) and part was used to prepare blood smears, which were fixed with absolute methanol for 3 min and stained with 10% Giemsa for 30 min (Eisen and Schall, 2000). The remaining blood was stored in 99.8% absolute ethyl alcohol PA of the Neon brand for molecular purposes. After blood collection, the animals were released in the same environment where they were captured.

The parasitic forms were observed under a Digilab DI-115 T light microscope (Digilab, Brazil) at magnifications of ×400 and ×1000. Images were captured using a digital camera and processed with ImageView® software (BestScope, China), with morphometric data (length, width and area) in micrometers (µm) presented as mean ± standard deviation and range (minimum–maximum). Parasitemia was determined by counting the number of parasites observed in 2000 erythrocytes, divided into 20 fields of 100 erythrocytes (Godfrey et al. 1987).

### DNA extraction, sequencing and phylogenetic analysis

Total DNA was extracted from blood samples using the DNeasy Blood and Tissue Kit (QIAGEN, Valencia, CA), following the manufacturer’s protocol. Parasite DNA detection by PCR (polymerase chain reaction) was performed using Hep300 (5’-GTT TCT GAC CTATCA GCT TTC GAC G-3’) and ER (5’-CTT GCG CCT ACT AGG CAT TC-3’) primers, which amplified an ≈1355 base pair (bp) fragment of the *18S* rDNA gene (Ujvari et al. 2004). The PCR amplifications were carried out in a final volume of 25 μL, containing 2.5 μL of 10 × PCR buffer, 1.5 μL of MgCl₂ (25 mM), 0.5 μL of each dNTP (10 mM), 0.5 μL of each primer (Hep300 and ER, 10 μM), 0.2 μL of Taq DNA polymerase (5 U/μL), 2 μL of template DNA, and nuclease-free water to complete the volume. The PCR consisted of an initial denaturation at 94 °C for 3 min, followed by 45 cycles of 94 °C for 45 s, 56 °C for 1 min, 72 °C for 40 s and a final extension at 72 °C for 10 min. Negative (ultrapure water) and positive (DNA of *Lankesterella* spp. previously amplified) controls were used in every run. To confirm the amplification of the targeted fragment, 2 μL of the final PCR product were run on 1.5% agarose gel. Samples presenting the target DNA size were considered positive. Amplicons were purified according to the manufacturer’s instructions using the Wizard® SV Gel and PCR Clean-Up System. PCR products were sequenced in both directions with the mentioned primers using the BigDye™ Terminator v.3.1 Cycle Sequencing Ready Reaction Kit (Applied Biosystems, Foster City, CA, USA) and the ABI 3100 Genetic Analyzer (Applied Biosystems, Foster City, CA, USA).

The quality of sequencing and sequences analysis was conducted in Geneious Prime 2023.2.1 (https://www.geneious.com). The obtained sequences were aligned to create a consensus sequence and compared to available sequences in GenBank. All sequences obtained in the present study were deposited in GenBank (accession numbers PX404938 and PX404939).

The sequence database for the Bayesian phylogenetic inference (BI) was constructed using Geneious Prime 2023.2.1 (https://www.geneious.com) and aligned using MAFFT software online v.7 (Katoh et al. 2018). The alignment consisted of 106 sequences up to 1355 bp. The *18S* rDNA sequences from *Plasmodium, Haemoproteus* and *Leucocytozoon* were used as an outgroup. The best-fit substitution model SYM + G was selected by MrModelTest2 software version 2.3 (Nylander, 2019). The BI was constructed with MrBayes version 3.2.7 (Ronquist et al. 2012). Each run was conducted with 4 chains and with a sampling frequency of every 100th generation over 3 million generations. We discarded 25% of the trees as ‘burn‐in.’ The remaining trees were used to construct a consensus tree, which was visualized using FigTree v.1.4.0 software (Rambaut, 2012). Using an alignment of 504 bp, the sequence divergence between all *Lankesterella* sequences from lizards was calculated with MEGA X: Molecular Evolutionary Genetics Analysis across computing platforms (Kumar et al. 2018), using a *p*-distance model, with uniform substitution rates.

## Results

Of the 5 green iguanas tested, 2 were positive for haemococcidian parasites by light microscopy and molecular screening for the 18S rDNA gene. Each infected host harboured a single haemococcidian sequence, with no evidence of mixed infections in the chromatograms. Sequencing results revealed 2 distinct genotypes belonging to the genus *Lankesterella*: genotype I (GenBank accession number PX404938) and genotype II (GenBank accession number PX404939). The intraspecific divergence (*p*-distance) between these 2 genotypes was 4.3% (Table 2).

**Table 2.** Genetic distance matrix (*p*-distance) based on the 504-bp fragment of the *18S* rDNA gene between *Lankesterella* sequences from lizard hosts. Diagonal cells represent self-comparisons. GenBank accession numbers and corresponding strain IDs are indicated. Sequences include newly obtained sequences from *Iguana iguana* (*Lankesterella nucleoflexa* sp. Nov. and *Lankesterella* sp.) And comparative sequences from GenBank Table 2 long description.

		1	2	3	4	5	6	7	8	9	10	11	12	13	14	15	16	17	18	19	20	21	22	23	24	25	26	27	28	29	30	31	32	33	34
**1**	MF167550 *Lankesterella* sp.																																		
**2**	MF167551 *Lankesterella* sp.	6.8%																																	
**3**	MF167553 *Lankesterella* sp.	7.4%	8.1%																																
**4**	KU180248 *Lankesterella* sp.	7.3%	7.1%	8.3%																															
**5**	OR425022 *Lankesterella desseri*	7.1%	7.9%	8.5%	4.2%																														
**6**	OR425019 *L. desseri*	7.3%	8.1%	8.7%	4.4%	0.2%																													
**7**	OR425032 *L. desseri*	7.5%	8.3%	8.9%	4.6%	0.4%	0.6%																												
**8**	OR425028 *L. desseri*	7.3%	8.1%	8.7%	4.4%	0.2%	0.4%	0.6%																											
**9**	MF167544 *Lankesterella* sp.	6.5%	7.7%	8.5%	3.2%	4.5%	4.7%	4.9%	4.7%																										
**10**	MF167552 *Lankesterella* sp.	6.5%	7.9%	8.5%	3.2%	4.0%	4.3%	4.5%	4.3%	0.4%																									
**11**	MF167555 *Lankesterella* sp.	6.1%	7.5%	8.1%	3.4%	4.3%	4.5%	4.7%	4.5%	0.6%	0.6%																								
**12**	MF167554 *Lankesterella* sp.	6.5%	6.9%	8.3%	3.6%	4.0%	4.2%	4.4%	4.2%	2.2%	2.2%	1.6%																							
**13**	KJ131417 *Lankesterella* sp.	7.1%	7.9%	8.7%	5.3%	5.5%	5.7%	5.9%	5.7%	5.1%	5.1%	4.5%	3.2%																						
**14**	MF167545 *Lankesterella* sp.	7.1%	7.9%	8.7%	4.8%	5.9%	6.1%	6.3%	6.1%	5.5%	5.5%	4.9%	5.5%	5.9%																					
**15**	MF167546 *Lankesterella* sp.	6.7%	7.7%	8.3%	4.4%	5.5%	5.7%	5.9%	5.7%	5.1%	5.1%	4.5%	5.1%	5.5%	0.8%																				
**16**	MF167549 *Lankesterella* sp.	7.1%	7.5%	7.9%	5.1%	6.1%	6.3%	6.5%	6.3%	5.7%	5.3%	5.3%	5.5%	5.9%	2.4%	2.2%																			
**17**	OR425021 *L. desseri*	7.6%	7.6%	9.8%	6.1%	7.0%	7.2%	7.0%	7.2%	5.7%	5.9%	5.3%	5.1%	6.5%	7.4%	7.0%	7.8%																		
**18**	OR425026 *L. desseri*	7.4%	7.4%	9.6%	5.9%	6.7%	7.0%	6.7%	7.0%	5.5%	5.7%	5.1%	4.9%	6.3%	7.2%	6.7%	7.6%	0.2%																	
**19**	OR425023 *L. desseri*	7.0%	7.0%	9.2%	5.5%	6.4%	6.6%	6.8%	6.6%	5.1%	5.3%	4.7%	4.5%	5.9%	6.8%	6.4%	7.2%	0.4%	0.2%																
**20**	**PX404938 *Lankesterella nucleoflexa* sp. nov.**	**8.5%**	**8.5%**	**10.1%**	**6.9%**	**7.1%**	**7.3%**	**6.7%**	**7.3%**	**6.1%**	**6.3%**	**5.7%**	**5.7%**	**7.1%**	**7.3%**	**6.9%**	**7.7%**	**2.9%**	**2.7%**	**2.7%**															
**21**	OR425015 *L. desseri*	8.1%	7.5%	10.5%	6.9%	7.5%	7.7%	7.1%	7.7%	6.7%	6.9%	6.3%	6.1%	7.9%	7.5%	7.3%	7.7%	3.7%	3.5%	3.5%	**3.7%**														
**22**	OR425016 *L. desseri*	8.3%	8.1%	11.0%	7.1%	7.7%	7.9%	7.3%	7.9%	6.9%	7.1%	6.5%	6.3%	8.1%	7.9%	7.7%	8.1%	3.9%	3.7%	3.7%	**4.3%**	0.6%													
**23**	OR425030 *L. desseri*	8.1%	7.9%	10.8%	6.9%	7.5%	7.7%	7.1%	7.7%	6.7%	6.9%	6.3%	6.1%	7.9%	7.7%	7.5%	7.9%	3.7%	3.5%	3.5%	**4.1%**	0.4%	0.2%												
**24**	OR425029 *L. desseri*	8.3%	7.9%	10.8%	6.7%	7.3%	7.5%	7.1%	7.5%	6.5%	6.7%	6.1%	5.9%	7.7%	7.5%	7.3%	7.7%	3.5%	3.3%	3.3%	**4.1%**	0.8%	0.6%	0.4%											
**25**	OR425024 *L. desseri*	8.1%	7.7%	10.5%	7.1%	7.7%	7.9%	7.3%	7.9%	6.9%	7.1%	6.5%	6.3%	8.1%	7.7%	7.5%	7.9%	3.9%	3.7%	3.7%	**3.4%**	0.2%	0.8%	0.6%	1.0%										
**26**	OR425017 *L. desseri*	8.1%	7.7%	10.5%	7.3%	7.9%	8.1%	7.5%	8.1%	7.1%	7.3%	6.7%	6.5%	7.9%	7.9%	7.7%	8.1%	3.9%	3.7%	3.7%	**3.7%**	0.4%	1.0%	0.8%	1.2%	0.2%									
**27**	OR425020 *L. desseri*	8.3%	7.7%	10.4%	7.3%	7.9%	8.1%	7.7%	8.1%	7.1%	7.3%	6.7%	6.5%	7.9%	7.9%	7.7%	7.7%	3.9%	3.7%	3.7%	**4.3%**	0.6%	1.2%	1.0%	1.4%	0.8%	0.6%								
**28**	**PX404939 *Lankesterella* sp.**	**7.7%**	**7.5%**	**10.6%**	**7.3%**	**7.3%**	**7.5%**	**6.9%**	**7.5%**	**6.7%**	**6.9%**	**6.3%**	**6.3%**	**8.1%**	**7.7%**	**7.5%**	**7.9%**	**4.3%**	**4.1%**	**4.1%**	**4.3%**	**0.6%**	**1.2%**	**1.0%**	**1.4%**	**0.8%**	**1.0%**	**1.2%**							
**29**	OR425025 *L. desseri*	8.5%	8.1%	11.2%	7.5%	7.5%	7.7%	7.1%	7.7%	6.7%	6.9%	6.3%	6.3%	8.1%	7.5%	7.3%	7.9%	3.7%	3.5%	3.5%	**3.4%**	1.4%	1.6%	1.4%	1.4%	1.6%	1.8%	2.0%	**2.0%**						
**30**	OR425018 *L. desseri*	8.5%	8.1%	11.2%	7.5%	7.5%	7.7%	7.1%	7.7%	6.7%	6.9%	6.3%	6.3%	8.1%	7.5%	7.3%	7.9%	3.7%	3.5%	3.5%	**3.4%**	1.4%	1.6%	1.4%	1.4%	1.6%	1.8%	2.0%	**2.0%**	0.0%					
**31**	**31** OR425027 *L. desseri*	8.5%	8.1%	11.2%	7.5%	7.5%	7.7%	7.1%	7.7%	6.7%	6.9%	6.3%	6.3%	8.1%	7.5%	7.3%	7.9%	3.7%	3.5%	3.5%	**3.4%**	1.4%	1.6%	1.4%	1.4%	1.6%	1.8%	2.0%	**2.0%**	0.0%	0.0%				
**32**	OR425031 *L. desseri*	8.7%	8.3%	11.4%	7.7%	7.7%	7.9%	7.3%	7.9%	6.9%	7.1%	6.5%	6.5%	8.3%	7.7%	7.5%	8.1%	3.9%	3.7%	3.7%	**3.7%**	1.6%	1.8%	1.6%	1.6%	1.8%	2.0%	2.2%	**2.2%**	0.2%	0.2%	0.2%			
**33**	MF167547 *Lankesterella* sp.	9.3%	8.6%	11.2%	7.1%	7.5%	7.7%	7.1%	7.7%	6.9%	7.1%	6.5%	6.3%	7.7%	7.3%	7.1%	7.9%	4.5%	4.3%	4.3%	**4.1%**	2.4%	3.0%	2.8%	2.9%	2.6%	2.8%	2.6%	**3.1%**	3.0%	3.0%	3.0%	3.3%		
**34**	MF167548 *Lankesterella* sp.	8.7%	7.9%	10.6%	6.7%	6.9%	7.1%	6.5%	7.1%	6.5%	6.7%	6.5%	6.1%	7.5%	7.3%	7.1%	7.7%	4.1%	3.9%	3.9%	**3.7%**	2.0%	2.6%	2.4%	2.4%	2.2%	2.4%	2.6%	**2.6%**	2.6%	2.6%	2.6%	2.8%	0.8%	

**Figure 2.**
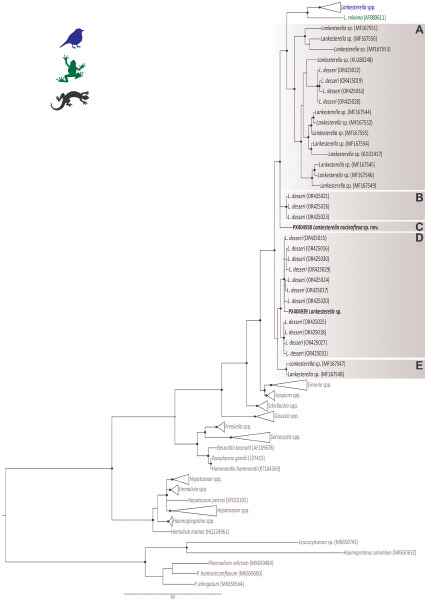
Bayesian inference tree of partial *18S* rDNA gene sequences (1355bp) of *Lankesterella* parasites. Haemosporidian parasite DNA sequences (*Plasmodium, Haemoproteus* and *Leucocytozoon*) were used as outgroup. GenBank accession numbers are indicated in parenthesis. *Lankesterella* sequences from lizards are indicated in different shades of brown. Sequences obtained in this study are given in bold. Nodes with posterior probability of <90% are marked with grey dots, and ≥90% are marked with black dots. Figure 2 long description.

The BI analysis based on the 18S rDNA gene placed the 2 *Lankesterella* sequences from this study in 2 distinct and well-supported clades (Figure 2). Genotype I (PX404938) was placed in a separate branch closely with *L. desseri* and other *Lankesterella* parasites from lizards (Figure 2, Clade C). The genetic distance within this clade ranged from 0.2% (primarily between *L. desseri* haplotypes) to 10.1% (between our genotype I and *Lankesterella* sp. MF167553). Genotype II (PX404939) clustered with 7 other haplotypes of *L. desseri*, which were closely related to 4 additional haplotypes of the same parasite (Figure 2, Clade D). In this clade, the genetic distance varied from 0.2% (mainly between *L. desseri* haplotypes) to 11% (between *L. desseri* – OR425016, and *Lankesterella* sp. – MF167553). Specifically, the genotype II (PX404939) showed a genetic distance ranging from 0.6% to 1.4% with the *L. desseri* haplotypes in the same clade (Table 2).

Based on an integrative analysis of phylogenetic positioning, molecular data and morphological characteristics, our findings support that the 2 distinct *Lankesterella* genotypes isolated from *I. iguana* were classified as 2 separate species, with genotype I formally described herein as the new taxon, *L. nucleoflexa* sp. nov., and genotype II designated as a *Lankesterella* sp.

*Species description*

Phylum Apicomplexa (Levine, 1970)

Class Conoidasida (Levine, 1988)

Order Coccidia (Leuckart, 1879)

Suborder Eimeriorina (Léger, 1911)

Family Lankesterellidae (Nöller, 1920)

Genus *Lankesterella* (Labbé, 1899)

**Figure 3.**
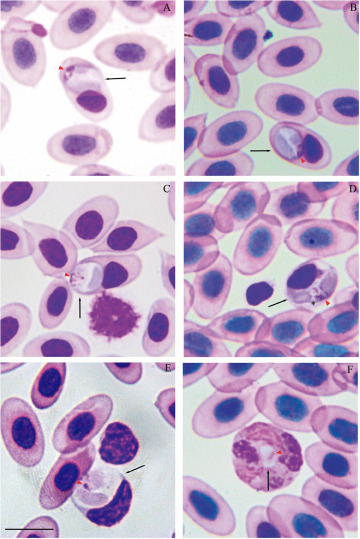
*Lankesterella nucleoflexa* sp. nov. infecting blood cells of *Iguana iguana*. (A–C) Intraerythrocytic sporozoites folded in spherical body. (D) Intraerythrocytic sporozoite with elongated body. (E) Sporozoite within a mononuclear leucocyte. (F) Sporozoite within a heterophil. Black arrows – parasites; red arrowheads – parasite nucleus; asterisk – refractile body. Thin blood smears stained with Giemsa. Scale bar = 10 μm. Figure 3 long description.

***Lankesterella nucleoflexa* sp. nov. (**Figure 3; Table 3)

**Table 3.** Morphometric characteristics of haemococcidian sporozoites detected in green iguana (*Iguana iguana*) from this study compared with other species described in lizards from the New World. Measurements are in micrometers (µm) and presented as mean ± standard deviation (SD) followed by the range (maximum and minimum values) Table 3 long description.

Parasite/Characteristic	*Lankesterella desseri*	*Lankesterella golvani*	*Lankesterella occidentalis*	*Schellackia landau*	*Lansonia iguanae*	*Lainsonia legeri*	*Lankesterella nucleoflexa* nov. sp.	*Lankesterella* sp.
	Chang et al. 2023^a^	Megía-Palma et al. 2017	Megía-Palma et al. 2017^b^	Lainson et al. 1976^b^	Landau, 1973^a^	Landau et al. 1974^b^	This study	
Intraerythrocytic sporozoite	59		47				14	7
Length	12.25 ± 1.62 (15.9−7.3)		7.3 ± 1.2	6.5 ± 0.4 (7.0−6.0)	11		13.60 ± 1.40 (15.76−11.43)	14.77 ± 0.88 (16.45−14.01)
Width	3.71 ± 0.84 (5.49−1.87)		3.5 ± 0.9	5.5 ± 0.4 (6.0−5.0)	7		3.00 ± 0.71 (4.53−1.83)	2.71 ± 0.22 (2.97−2.33)
Area							35.59 ± 7.83 (48.48−18.47)	32.39 ± 3.69 (35.44−24.47)
Nucleus length							3.13 ± 0.77 (4.58−1.48)	1.93 ± 0.47 (2.53−1.33)
Nucleus width							2.39 ± 0.99 (4.64−0.80)	2.66 ± 0.31 (3.05−2.17)
Nucleus area							6.24 ± 2.33 (11.42−2.81)	5.89 ± 1.20 (7.45−4.47)
Infected erythrocyte							14	7
Length							15.49 ± 1.45 (18.49−12.72)	14.46 ± 1.20 (16.10−11.29)
Width							8.98 ± 0.47 (9.87−8.41)	7.87 ± 0.98 (9.46−5.93)
Area							113.21 ± 14.83 (146.67−85)	90.33 14.44 (112.39−67.04)
Nucleus length							5.09 ± 0.97 (6.68−3.59)	5.32 ± 0.86 (6.58 − 3.98)
Nucleus width							6.29 ± 0.97 (6.68−3.39)	5.25 ± 0.39 (6.30−4.67)
Nucleus area							24.59 ± 5.16 (35.88−15.26)	21.36 ± 2.52 (25.92−17.08)
Uninfected erythrocyte							20	20
Length							14.24 ± 0.99 (15.77−11.59)	14.87 ± 1.13 (16.40−12.08)
Width							8.30 ± 0.88 (10.30−6.47)	8.26 ± 1.13 (11.32−6.15)
Area							93.27 ± 11.30 (115.23−71.77)	98 ± 15.91 (121.28−68.46)
Nucleus length							4.69 ± 0.50 (5.58−3.98)	4.64 ± 0.55 (6.22−3.81)
Nucleus width							6.44 ± 0.80 (8.23−5.14)	6.55 ± 0.59 (8.23−5.61)
Nucleus area							24.34 ± 4.21 (32.19−17.38)	24.22 ± 4.14 (32.98−17.72)
Intraleukocytic sporozoites			57					
Length			9.3 ± 1.6	9.5 ± 0.5 (10.0−9.0)		9.5		
Width			4.2 ± 1.2	5.5 ± 0.4 (6.0−5.0)		4.0		
Area								
Intraleukocytic (monocyte) sporozoite							5	27
Length							19.13 ± 2.48 (20.93−14.77)	16.93 ± 1.89 (21.88−13.87)
Width							4.00 ± 0.23 (4.31−3.69)	3.82 ± 0.68 (4.96−2.44)
Area							71.72 ± 15.83 (99.30−59.23)	60.86 ± 19.24 (108.20−30.39)
Nucleus length							4.49 ± 0.51 (5.08−3.68)	3.36 ± 0.94 (6.69−1.91)
Nucleus width							3.28 ± 0.51 (4.04−2.81)	2.57 ± 0.67 (4.27−1.61)
Nucleus area							10.80 ± 1.93 (12.69−7.76)	8.07 ± 2.82 (15.50−3.26)
Infected monocyte							5	27
Length							13.89 ± 2.71 (18.22−11.74)	11.97 ± 2.26 (18.30−8.84)
Width							12.06 ± 0.76 (12.91−11.05)	10.31 ± 2.01 (14.47−6.18)
Area							131.17 ± 24.92 (175.09−116.60)	96.31 ± 31.99 (182.45−53.07)
Nucleus length							13.56 ± 4.13 (16.05−6.30)	8.02 ± 1.59 (10.50−4.15)
Nucleus width							5.83 ± 3.53 (12.14−4.05)	4.92 ± 2.74 (16.17−2.53)
Nucleus area							50.39 ± 5.79 (60.63−46.83)	29.65 ± 9.15 (52.67−18.74)
Uninfected monocyte							20	20
Length							11.75 ± 3.18 (18.49−7.52)	10.38 ± 0.82 (12.44−9.17)
Width							9.94 ± 1.63 (11.8−7.35)	9.93 ± 0.83 (11.84−8.21)
Area							95.28 ± 39.89 (175.74−44.67)	86.33 ± 14.56 (109.71−65.39)
Nucleus length							8.37 ± 2.27 (11.29−4.15)	6.80 ± 1.16 (9.19−4.82)
Nucleus width							8.57 ± 1.45 (11.65−6.90)	8.93 ± 1.40 (11.50−5.37)
Nucleus area							60.86 ± 17.91 (87.86−32.33)	53.59 ± 2.90 (59.27−48.97)
Intraleukocytic (heterophil) sporozoite		21					3	2
Length		8.4 (7.1−9.9)					4.54 ± 1.21 (5.47−3.03)	16.58 ± 4.14 (19.53−13.63)
Width		3.9 (2.6−4.5)					2.74 ± 0.49 (3.06−2.18)	3.81 ± 0.94 (4.48−3.14)
Area							9.72 ± 3.61 (11.81−5.55)	46.76 ± 0.34 (47.01−46.52)
Nucleus length							1.61 ± 0.20 (1.81−1.42)	3.52 ± 1.49 (4.58−2.57)
Nucleus width							0.83 ± 0.22 (1.06−0.63)	3.62 ± 0.52 (3.99−3.25)
Nucleus area							1.41 ± 0.33 (1.78−1.17)	11 ± 3.06 (13.17−8.83)
Infected heterophil							3	2
Length							15.15 ± 4.08 (19.01−10.87)	16.40 ± 1.03 (17.12−15.67)
Width							9.70 ± 3.69 (7.32−6.64)	13.50 ± 2.18 (15.04−11.95)
Area							117.83 ± 55.28 (169.03−59.21)	171.10 ± 31.98 (193.71−148.48)
Nucleus length							6.26 ± 0.63 (6.69−5.72)	6.54 ± 0.83 (7.12−5.95)
Nucleus width							10.87 ± 3.5 (14.73−7.9)	7.83 ± 0.30 (8.04−7.61)
Nucleus area							39.25 ± 16.07 (57.41−26.84)	42.24 ± 10.13 (49.41−35.08)
Uninfected heterophil							10	10
Length							17.68 ± 2.31 (20.59−13.62)	17.65 ± 1.37 (19.50−15.10)
Width							13.26 ± 1.76 (16.66−10.66)	13.42 ± 1.27 (15.20−11.25)
Area							160.08 ± 30.58 (215.96−121.60)	158.30 ± 19.84 (190.44−126.34)
Nucleus length							4.83 ± 0.59 (5.83−4.18)	4.90 ± 0.48 (5.67−4.25)
Nucleus width							13.63 ± 3.67 (20.38−6.68)	12.78 ± 3.05 (18.33−8.92)
Nucleus area							55.06 ± 10.91 (73.95−34.54)	50.26 ± 9.21 (66.70−38.95)
Free sporozoite			6					8
Length			8.6 ± 1.6	13.0				18.47 ± 2.28 (21.65−15.01)
Width			2.9 ± 0.5	3.0				3.07 ± 1.07 (4.59−1.96)
Area								47.74 ± 12.73 (65.54−33.69)
Nucleus length								2.88 ± 1.25 (4.80−1.52)
Nucleus width								3.30 ± 0.81 (5.08−2.71)
Nucleus area								6.99 ± 2.61 (12.10−3.29)

a Authors did not provide morphometric data for sporozoites that infect leucocytes.

b Authors did not morphometrically differentiate sporozoites in different white blood cells; the values were grouped into intraleukocytic sporozoites.

Type host: Green iguana *Iguana iguana* (Linnaeus, 1758) (Iguanidae)

Other hosts: Unknown.

Type locality: Cerrado forest fragment, Ramal Antena 1, municipality of Macapá, Amapá, Brazil (0°00’53.9” S, 51°04’34.2” W).

Other locality: Unknown.

Site of infection: Blood erythrocytes and leucocytes (mononuclear leucocytes and heterophils).

Prevalence: One individual of *Iguana iguana* was infected.

Parasitaemia: Parasitaemia was 54 parasites for every 2,000 erythrocytes (2.7%).

Vector: Unknown.

Etymology: The specific epithet ‘*nucleoflexa*’ is derived from Latin, where ‘*nucleo*’ refers to the parasite’s nucleus, and ‘flexa’ means ‘flexible’. This name highlights the distinctive characteristic of the parasite’s nucleus.

Type material: hapantotype (1 blood slides) from *Iguana iguana* were deposited at the Hemoparasite Collection of the Federal University of Minas Gerais (Coleção de Hemoparasitos da Universidade Federal de Minas Gerais – UFMG-HEM) (UFMG-HEM-0055).

DNA sequences: The *18S* ribosomal DNA gene sequences were deposited in the GenBank database under accession number (GenBank Accession number PX404938).

ZooBank registration: The Life Science Identifier (LSID) for *L. nucleoflexa* sp. nov. is urn:lsid:zoobank.org:act:1B451F2D-CF00-4377-B4DD-9443B796FB80.

Diagnosis: Sporozoites were found mainly in erythrocytes (70.37%; *n* = 38/54) and less frequently in leucocytes (24.1%; *n* = 13/54) (mononuclear leucocytes and heterophils). Sporozoites are commonly folded into a large spherical body when infecting erythrocytes and mononuclear leucocyte (Figures 3A–C) and can be rarely seen in their typical elongated form when infecting erythrocytes (Figure 3D). In heterophils, sporozoites exhibit bean-shaped forms and are smaller than those observed in erythrocytes and mononuclear leucocytes (Figure 3F; Table 3). Erythrocytic sporozoites have unequal ends, 1 rounded and the other slightly pointed, while leukocytic parasites present both rounded ends. All sporozoite forms have pale cytoplasm, with a uniform bluish-grey hue. In spherical forms, a colourless cleft is visible between the 2 ends of the sporozoite, and the cytoplasm often exhibits a denser periphery, outlining the parasite. Sporozoite nuclei stain pink and vary in shape and position, from dispersed chromatin filaments (Figure 3D) and condensed masses (Figures 3A and B). Usually, parasite nuclei in erythrocytes appear as condensed masses positioned towards one end. In leucocytes, they are often band-like, centrally positioned and typically occupy the entire width of the host cell. In this case, the band-like structure is observed only in heterophils. In erythrocytes, sporozoites are in a polar position, displacing the host cell nucleus to the cell margin (Figures 3A–D). Sporozoites infecting mononuclear leucocytes are larger than erythrocytic forms (Table 3), occupying the entire cytoplasm of the host cell, and deforming their nuclei (Figure 3D). In heterophils, sporozoites are in the central region of the host cell and do not produce any visible effect in the host cells or their nuclei (Figure 3F; Table 3). Free sporozoites were not observed. A single refractile body is variably present in elongated forms and is inconspicuous in spherical and bean-shaped parasites. The refractile body has a small, round shape and, when present, is usually located close to the parasite’s nucleus (Figure 3D).

Effects on the host cell: In erythrocytes and mononuclear leucocyte, sporozoites produce visible effects. Parasitized erythrocytes became slightly hypertrophied and sometimes distorted, with their nuclei distorted and displaced towards one of the extremities of the host cell (Figure 3E; Table 3). Infected mononuclear leucocytes were also hypertrophied with their nuclei strongly deformed and pushed to one of the margins of the host cell (Figure 3D; Table 3). No deformation was seen in infected heterophils.

**Figure 4.**
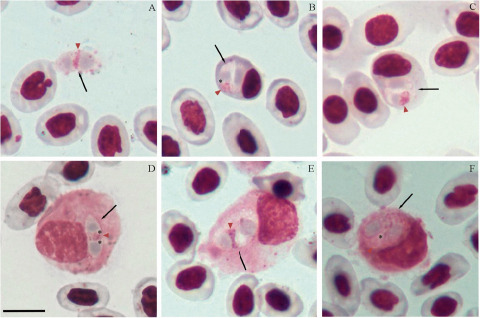
*Lainsonia iguanae* in green iguana (*Iguana iguana*) from Para, Brazil, voucher material (no. 15 HA) from Landau’s collection, MNHN. (A) Free sporozoite. (B-C) Intraerythrocytic sporozoite. (D–F) Sporozoites within leucocytes. Black arrows – parasites; red arrowheads – parasite nucleus; asterisk – refractile body. Thin blood smears stained with Giemsa. Scale bar = 10 μm. Figure 4 long description.

Remarks: Landau (1973) morphologically described *L. iguanae* in hosts captured in Brazil (states of Pará and Pernambuco) and French Guiana (Cayenne), thus being the first haemococcidian described infecting this host (Figure 4). Later, this parasite species was also found by Telford (1977) in the same host from Panama and Venezuela, but the author did not provide morphological data for the parasite found. In the original description, Landau (1973) stated that similar sporozoites were observed in erythrocytes and monocytes of hosts from the 3 localities (Pará, Pernambuco and Cayenne). However, the characterization of the species and its development in the experimental vector (*Aedes aegypti* Linnaeus, 1762) was based on the parasites found in a single host collected in Belém, state of Pará, Brazil. Furthermore, the description provided for the sporozoites of *L. iguanae* in the peripheral blood was quite vague. This limits the comparison with *L. nucleoflexa* sp. nov. because, beyond the unknown intraspecific morphological variation in *L. iguanae* that could include forms similar to *L. nucleoflexa* sp. nov., it is impossible to determine if the other hosts in the other localities harboured *L. iguanae* or another haemococcidian species. Still, we consider it important to compare the 2 species, since there is no published data for the *L. iguanae* leukocytic forms, we will be limited to the erythrocytic sporozoites. Sporozoites of *L. iguanae* differs from *L. nucleoflexa* sp. nov. by being smaller (Table 3), having both ends rounded, and a triangular nucleus positioned between 2 refractile bodies (Figure 4). Compared to other described species of lizards from the New World, *L. legeri, S. landauae, L. golvani, L. occidentalis* and *L. desseri,* the sporozoites of *L. nucleoflexa* sp. nov. are morphologically different (Table 2). Sporozoites of *L. legeri* only infect monocytes and lymphocytes; despite being surrounded by a clear space not limited by a membrane inside the host cell, they are always elongated instead of folded like *L. nucleoflexa* sp. nov. Additionally, they exhibit a smaller length than *L. nucleoflexa* sp. nov. and possess a transverse or triangular nucleus (Landau et al. 1974; Table 2). For *S. landauae*, sporozoite morphology varies depending on the host cell, which can be leucocytes (monocytes or lymphocytes) and erythrocytes. Within leucocytes assume a stumpy bean or sausage shape and possess a smaller length and bigger width than *L. nucleoflexa* sp. nov. (Lainson et al. 1976; Table 2). In erythrocytes, they are folded up into a tight oval body with smaller dimensions than *L. nucleoflexa* sp. nov. (Lainson et al. 1976; Table 2). The sporozoites described for *L. golvani* and *L. occidentalis* are elongated and do not exhibit a spherical, folded shape, unlike those of *L. nucleoflexa* sp. nov. (Bonorris and Ball, 1955; Megía-Palma et al. 2017; Table 2). Furthermore, *L. golvani* exclusively infects leucocytes (monocytes, lymphocytes and primarily heterophils) without deforming the host cells (Telford, 2009). For *L. desseri,* sporozoites varied in shape, ranging from bent to lentiform, and most frequently fusiform, displaying eosinophilic and granular cytoplasm, and slightly displacing or distorting both the host cell and its nuclei (Chang et al. 2023).

**Figure 5.**
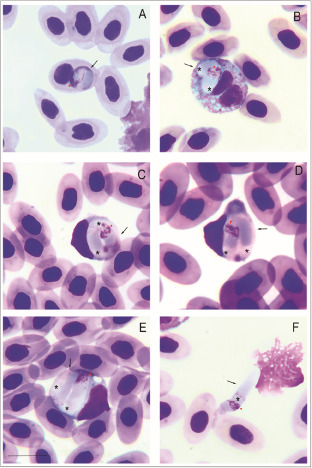
*Lankesterella* sp. infecting blood cells of *Iguana iguana*. (A) Intraerythrocytic sporozoite. (B) Small sporozoite in a heterophil. (C–D) Large sporozoites in mononuclear leucocytes. (F) Free sporozoite. Black arrows – parasites; red arrowheads – parasite nucleus; asterisks – refractile bodies. Thin blood smears stained with Giemsa. Scale bar = 10 μm. Figure 5 long description.

***Lankesterella* sp. (PX404939; UFMG-HEM-0056;** Figure 5; Table 3)

This haemococcidian parasite is characterized by sporozoites infecting mainly in mononuclear leucocyte (59.6%; *n* = 42/70), and less frequently in erythrocytes (12.8%; *n* = 9/70) and heterophils (2.8%; *n* = 2/70) of *I. iguana* in Brazil (0°00’59.3” S, 51°04’43.6” W).

Within host cells, sporozoites are typically curved, occasionally with rounded ends (Figure 5A), with rounded ends. The cytoplasm stains bluish-grey and has a heterogeneous appearance, usually showing reddish-staining granules (Figure 5C). Eventually, a colourless slit of variable size is visible between its ends (Figures 5C and 3D). The nuclei of the parasites are large, with irregularly shaped chromatin, and located centrally or towards one of the ends (Figures 5A and 5B). Sporozoites infecting mononuclear leucocytes are large and wide, occupying the entire host cell cytoplasm, which severely distorts the host cell and its nucleus (Figure 5D; Table 2). The parasite is closely appressed to the host cell nucleus pushing it towards the opposite pole and markedly deforming the host cell nucleus when infecting mononuclear leucocytes Free sporozoites were common (Figure 5F; Table 3), exhibiting slender and elongated bodies, with one pointed end and the opposite rounded. Sporozoites possess 1 or 2 spherical refractile bodies located in variable positions. The presence of the refractile bodies is constant in free forms and sporozoites within host cells. Sporozoites produce visible effects in erythrocytes and mononuclear leucocyte (Figures 5B–D; Table 3). Parasitized mononuclear leucocyte exhibits the most pronounced changes, with marked hypertrophy and nuclear deformations. Erythrocytes show increased volume and distortions, with their nuclei pushed toward the cell margin.

Remarks: This parasite clusters within a clade of *L. desseri* (Clade D; Figure 2), described by Chang et al. (2023), with minimal intergenotypic divergence. While this phylogenetic position suggests it may represent the same species, the observed polyphyletic position of the sequences named as *L. desseri* (Clades A, B and D; Figure 2), along with the lack of detailed morphological data from its previous descriptions (Chang et al. 2023), precludes a definitive comparison with the *Lankesterella* sp. from our study and its formal description as a new species. Further morphological data (e.g. on merogony, gamogony and sporogony) and additional molecular markers (e.g. mitochondrial gene amplification) are necessary to resolve this taxonomic conflict. Despite these uncertainties, it is valuable to compare its morphology to that of *L. nucleoflexa* sp. nov., also described in this study. Although both parasites have similar sporozoite shapes and infect the same type of host cells, they can be clearly distinguished. The sporozoites of *L. nucleoflexa* sp. nov. are typically spherical with a light cytoplasm, while those of the *Lankesterella* sp. genotype II are large and elongated, with a heterogeneous and granular cytoplasm (Figures 3 and 4; Table 3).

## Discussion

To our knowledge, this is the first study to employ an integrative approach, by combining phylogenetic analyses based on the *18S* rDNA gene and morphological data, to characterize haemococcidians infecting *I. iguana*, and as a result, we described *L. nucleoflexa* sp. nov. Furthermore, by presenting a complete and updated checklist of haemococcidian species from lizards, we highlight the existence of a significant gap in *Lankesterella* research in these hosts, particularly in terms of molecular characterization, which is likely among the most neglected within the phylum Apicomplexa, with over 40 years since the last species description in Brazil (see Table 1).

Previously, only one haemococcidian species had been described in green iguanas, *L. iguanae*, which Landau identified in 1973 through the morphology of the blood and tissue stages. Despite some erythrocytic forms observed in *L. nucleoflexa* sp. nov. and exhibiting characteristics that could be somewhat similar to *L. iguanae* (e.g. a spherical shape), other morphological and morphometric characteristics of the sporozoites reveal significant divergences from the species described by Landau, indicating that, *L. nucleoflexa* sp. nov. is distinct from *L. iguanae* (see remarks). Unfortunately, our study did not target other developmental stages (e.g. tissue stages) of *Lankesterella*, preventing a detailed comparison with this species.

By definition, *Lainsonia* differs from other haemococcidian genera by having merogony, gamogony and sporogony in the reticuloendothelial system, and sporozoites parasitizing erythrocytes or leucocytes (Landau, 1973; Landau et al. 1974; Lainson et al. 1976). However, these characteristics may not be sufficient to distinguish this genus, as some authors have, over the years, synonymized *Lainsonia* with *Schellackia*; both produce 8 sporozoites per oocyst, whereas *Lankesterella* oocysts produce 32 or more sporozoites (Levine, 1988; Megía-Palma et al. 2017). Although the parasites we found were morphologically similar to *Lainsonia*, their sequences grouped with other lineages from the genus *Lankesterella*. This finding is particularly interesting because it indicates that *Lainsonia* may be a synonym of *Lankesterella*, thus refuting its synonymy with *Schellackia*. Additionally, our data support the hypothesis that the genus *Lankesterella* in lizard hosts has a distribution restricted to the New World (Megía-Palma et al. 2017). To resolve the persistent taxonomic conflict surrounding the genus *Lainsonia*, more comprehensive morphological and molecular data are needed. Our findings highlight that the developmental characteristics previously used to classify haemococcidian genera, along with the overall systematics of the group, require a thorough review (Megía-Palma et al. 2014).

Other records of haemococcidians in *I. iguana* are scarce and generally not formally described. Telford (1977) mentioned a low prevalence of *L. iguanae* in green iguanas from Venezuela (*n* = 2/69) and Panama (*n* = 2/29), though without further descriptive detail. Similarly, morphotypes suggestive of haemococcidians were observed in captive iguanas in the USA (Harr et al., 2001). In Brazil, the most recent report was made by Lima et al. (2020) in green iguanas from Santarém, Pará state, where intraerythrocytic sporozoites were consistent with the genus *Lainsonia*, but without morphological descriptions or molecular support. Thus, this study significantly expands the knowledge of the geographic distribution of these parasites in Brazil, including new records in Eastern Amazonia, and provides the first molecular data associated with haemococcidians in green iguanas.

It is important to mention that our literature survey on the haemoparasite species of *I. iguana* uncovered descriptions of haemogregarines, specifically *H. iguanae* and *H. sinimbui* (Laveran and Nattan-Larrier, 1912; Carini, 1942). Their morphological characteristics bear a striking resemblance to haemococcidians previously described in these hosts (Landau, 1973; this study). *Hepatozoon iguanae,* whose type locality was addressing as South America, was observed infecting both erythrocytes and leucocytes and exhibited 3 distinct morphologies. These included small, often curved forms (5–6 × 1–2 μm) with one rounded and one tapered end, surrounded by a clear space. Larger parasites were noted, folded into spherical shapes (8–10 × 1–40 μm) with rounded or slightly tapered ends. Additionally, free forms were elongated (9–15 × 1 µm) and slightly curved, with rounded or sometimes tapered ends (Laveran and Nattan-Larrier, 1912). In contrast, *H. sinimbui*, discovered in hosts from Porto Nacional, Tocantins state and Brazil, was exclusively found in erythrocytes. This species was characterized by small, rounded forms (6–8 μm diameter) with a distinct line separating the 2 ends of the parasite internally, alongside free parasites that were elongated or banana-shaped (14 × 5 µm) (Carini, 1942). While neither description explicitly mentions the presence of refractile bodies or vacuoles, the available plates clearly illustrate that their cytoplasm was granular. Notably, in *H. sinimbui*, there was a distinct, clearer and rounder space near the parasite’s nucleus, which closely resembles refractile bodies (Carini, 1942). We acknowledge the possibility that these parasites are indeed haemogregarines, especially given that some *Hepatozoon* species infect reptile leucocytes (Morais et al. 2024). However, based on their unique morphology, we hypothesize they may in fact be haemococcidians.

From an ecological perspective, the hypothesis that these parasites are haemococcidians becomes quite plausible. *Iguana iguana* is an herbivorous species (Alberts et al. 2004), which makes their parasites inclusion among haemogregarines unlikely, as the latter’s life cycle typically involves the predation of infected paratenic vertebrate hosts (Smith, 1996) – a dietary habit not characteristic of green iguanas. In contrast, passive transmission through ingestion or bites from invertebrates, such as mites and mosquitoes, aligns well with the ecology of green iguanas and known life cycle of haemococcidians (Landau, 1973; Desser, 1993). Notably, Laveran and Nattan-Larrier (1912) reported an abundant presence of mites (*Amblyomma dissimile* Koch, 1844) on the examined hosts, further bolstering the haemococcidian hypothesis. Furthermore, the wide geographical distribution and high population densities of iguanids favour the persistence and diversification of haemococcidian lineages across different ecological regions (Campos and Desbiez, 2013; Maurer et al. 2020). This explains the observation of multiple morphotypes, even within single samplings, which is consistent with the high genetic diversity seen in haemococcidians (Megía-Palma et al. 2017). While definitive identification without molecular data remains challenging, the combined morphological, ecological and comparative evidence strongly suggests that the species described by Laveran and Nattan-Larrier (1912) and Carini (1942) belong to the *Lainsonia–Lankesterella* complex.

The life cycle of the 2 *Lankesterella* species described in this study remains unknown. Landau (1973) experimentally described the life cycle of *L. iguanae* in *I. iguana* following infection of *Aedes aegypti* mosquitoes. These vectors developed infections in stomach epithelial cells, but subsequent transmission to other lizard species was unsuccessful, suggesting vertebrate host specificity. Other studies also indicate that mosquitoes of the genus *Culex* and sandflies can act as vectors for different haemococcidians in lizards, promoting oocyst development and the release of infective sporozoites (Landau et al. 1974; Lainson et al. 1976; Klein et al. 1988). Furthermore, other invertebrates, such as mites, may play a role in *Lankesterella* transmission (Bonorris and Ball, 1955; Jordan and Friend, 1971), though this still needs more experimental confirmation (Megía-Palma et al. 2017).

Recent studies suggest high genetic diversity within *Lankesterella*, particularly in Neotropical reptiles (Megía-Palma et al. 2017; Er-Rguibi et al., 2023; Hajiyan and Javanbakht, 2022). This pattern is corroborated by our results, in which a relatively small number of hosts sampled in a limited area yielded 2 distinct genotypes of haemococcidians. In fact, *L. nucleoflexa* sp. nov. and *Lankesterella* sp. exhibit a genetic distance of 4.1%, which, combined with their distinct phylogenetic positions and morphological traits, reinforces their separation as valid and independent species.

Our phylogenetic analyses, based on the *18S* rDNA region, recover patterns consistent with previous haemococcidian phylogenies (Megía-Palma et al. 2014; 2017). These analyses reinforce the polyphyly of the family Lankesterillidae and the separation of *Lankesterella* clades among lineages obtained from birds, anurans and lizards (Megía-Palma et al. 2014; 2016, 2017; Chagas et al. 2021; Veith et al. 2023).

The 2 novel *Lankesterella* genotypes isolated from *I. iguana* show distinct phylogenetic positions and molecular divergences. Genotype I, described here as *L nucleoflexa* sp. nov., occupies an isolated branch, consistent with interspecific differentiation. This lineage is clearly separated from all others, including the clades A, B and D that contain sequences of *L. desseri* (Chang et al. 2023), with divergence values ranging from 2.7% to 8.5%. This strong divergence supports its recognition as a new species. In contrast, the genotype II, identified here as a *Lankesterella* sp., clustered within clade D, which comprises 7 other sequences identified as *L. desseri* (Chang et al. 2023).

These findings address the polytomy observed in *L. desseri* and supports the hypothesis that it is a polyphyletic taxon, comprising multiple genetically distinct species-level lineages that lack a single common ancestor (see Figure 2; Chang et al. 2023). While the internal divergence within the clade D is low (0.6–1.4%), a range compatible with intraspecific variation, the divergence between D and the other *L. desseri* clades A and B exceeds 6%. This high divergence is well above commonly applied thresholds for species delimitation in Apicomplexa using the 18S rDNA marker (Megía-Palma et al. 2014; 2016, 2017) and is consistent with the high intergenotypic divergence (up to 8%) found by Chang et al. (2023).

This combination of low intra-clade and high inter-clade divergence confirms that the subclades currently grouped under *L. desseri* likely represent multiple, distinct species. Therefore, while our study formally describes *L. nucleoflexa* sp. nov., we refrain from formally describing *Lankesterella* sp. as a new species, until a comprehensive taxonomic revision of the *L. desseri* complex is conducted. This revision will require expanded geographic sampling, detailed morphological data and higher-resolution molecular markers, such as mitochondrial genes.

The description of *L. nucleoflexa* sp. nov. expands our knowledge regarding the diversity of haemococcidians in Neotropical reptiles, particularly in iguanas from the Eastern Amazonia region, representing the first study with molecular characterization of haemococcidians in lizards from Brazil. Future studies should address the life cycle of these species, with vector incrimination and endogenous development in the tissues of the green iguana. Here, we also highlight the importance of integrative approaches, careful morphological comparisons and molecular analyses for the precise taxonomic delimitation of parasites with conserved morphology. These findings open new perspectives for more specific investigation with haemococcidians, highlighting the relevance of systematic studies in tropical regions that are still poorly explored.

## Data Availability

Sequence data are available at GenBank accessions: PX404938 and PX404939 (partial *18S* rDNA). Haemococcidan hapantotype and voucher specimens are deposited at the Coleção de Hemoparasitos da Universidade Federal de Minas Gerais (UFMG-HEM-0055 and UFMG-HEM-0056).

## Table 1 Long description

The table lists haemococcidian parasite species reported from lizards, pairing each parasite with its lizard host, any available partial 18S rDNA GenBank accession numbers, collection localities, and literature sources. Most records report no GenBank accession (“N/A”), indicating many occurrences are based on published observations rather than deposited sequences. The broadest host coverage is for Lankesterella golvani, recorded across many Anolis species from the Caribbean and the USA, with a single accession (KU180248) for Anolis carolinensis. Sequence accessions are provided for Lankesterella desseri in Ctenosaura similis from Nicaragua (OR425015–OR425032) and for Lankesterella occidentalis and Uta stansburiana in the USA (MF167550–MF167553). Schellackia records span Africa, the Middle East, Europe, and Asia, including accessions for S. bolivari (KJ131415–KJ131416) and S. orientalis (KJ131414), while many other Schellackia entries lack accessions. Localities are often multi-country for a single parasite–host pairing (for example, Lainsonia iguanae in Iguana iguana across several Neotropical countries), so the table summarizes reported presence rather than prevalence or intensity. Blank parasite cells indicate additional host rows continuing the same parasite species listed immediately above.

Navigate back to Table 1.

## Figure 1 Long description

The map shows the Macapá municipality in the State of Amapá, Eastern Amazonia, Brazil, with sampling sites for green iguanas marked by yellow circles. The left panel includes a regional map with a highlighted area for Macapá and a legend indicating 'Sampling site' with yellow circles, 'Amapá' in grey and 'Macapá' in orange. The right panel is a satellite image detailing urban and forested areas, with two yellow circles indicating sampling points. The reference system is SIRGAS 2000, UTM Zone 22, with data from IBGE (2004) and satellite imagery from Geoeye and Esri. The map includes scale bars for distance and coordinates for precise location identification.

Navigate back to Figure 1.

## Figure 2 Long description

The diagram is a Bayesian inference tree of partial rDNA gene sequences, oriented vertically from top to bottom. It illustrates the evolutionary relationships among various parasites, with sequences from lizards indicated in different shades of brown. The tree is divided into five main clades labeled A to E. Clade A includes Lankesterella sp. and Lankesterella desseri, with sequences such as MF167553 and MF167554. Clade B features L. desseri sequences like OR425016 and PX404939. Clade C includes sequences from L. desseri and Lankesterella sp. Clade D contains L. desseri sequences, including PX404938. Clade E features Lankesterella sp. MF167552. Nodes with posterior probabilities of less than 90 percent are marked with grey dots, while those with 90 percent or more are marked with black dots. The tree also includes outgroup sequences from Haemosporidian parasites, such as Plasmodium and Haemoproteus, located at the bottom. GenBank accession numbers are indicated in parentheses next to each sequence. The tree visually represents genetic distances and evolutionary relationships among the sequences, with branches indicating divergence points and sister group relationships.

Navigate back to Figure 2.

## Figure 3 Long description

The image A showing multiple oval cells with pale pink cytoplasm and dark purple oval nuclei on a light background. A thin black arrow points toward a small purple structure near the edge of one cell. The image B showing multiple oval cells with pale pink cytoplasm and dark purple oval nuclei on a light green background. A short black scale bar is near the lower center. A thin black arrow points to a round, pale area within one cell, with a small red arrowhead near the lower right of that cell. The image C showing multiple oval cells with pale pink cytoplasm and dark purple oval nuclei on a light background. A thin black arrow points to a pale round area within one cell. A small red arrowhead is near the upper left edge of that pale area. A dense dark purple cluster is present near the lower right. The image D showing multiple oval cells with pale pink cytoplasm and dark purple oval nuclei on a light green background. A thin black arrow points to a pale round area within one cell. Two small red arrowheads are placed near the right side of that cell. The image E showing multiple oval cells with pale pink cytoplasm and dark purple oval nuclei on a light background. A larger, darker purple round cell is near the center-right. A thin black arrow points toward a pale round area within a nearby cell. The image F showing multiple oval cells with pale pink cytoplasm and dark purple oval nuclei on a light green background. A larger round cell with darker purple material is near the center. A thin black arrow points upward toward the lower part of this larger cell.

Navigate back to Figure 3.

## Figure 4 Long description

The image A showing a pale background with several oval cells containing dark purple oval centers. Near the center, a light pink irregular area is marked by a black arrow pointing upward from below. Two small red arrowheads point toward the light pink area. The image B showing multiple oval cells with dark purple oval centers on a pale background. A black arrow points diagonally toward a light pink area near the center. Two red arrowheads point toward the same light pink area. The image C showing several oval cells with dark purple oval centers on a pale background. A black arrow points horizontally toward a small light pink area near the lower center. Two red arrowheads point toward the light pink area. The image D showing a large round pink cell occupying much of the frame, with a darker pink region inside it. A black arrow points diagonally toward a small cluster of dark dots near the right side of the large cell. Two red arrowheads point toward the same cluster. A black scale bar is present at the lower left. The image E showing multiple oval cells with dark purple oval centers on a pale background. Near the center, a light pink irregular area is present. A black arrow points upward toward this area from below. Two red arrowheads point toward the light pink area. The image F showing a large round pink cell near the center-right with a darker pink region inside it. A black arrow points diagonally toward a small cluster of dark dots near the upper left edge of the large cell. Two red arrowheads point toward the same cluster.

Navigate back to Figure 4.

## Figure 5 Long description

The image A showing many oval cells with dark purple oval centers on a pale background. One larger pale cell near the center contains several small dark inclusions marked by asterisks. The image B showing oval cells with dark purple centers. A larger pale cell contains clustered dark inclusions marked by asterisks. The image C showing oval cells with dark purple centers. A larger pale cell contains multiple dark inclusions marked by asterisks. The image D showing oval cells with dark purple centers. A larger pale cell contains several dark inclusions marked by asterisks. A small black arrow points toward the larger pale cell. The image E showing oval cells with dark purple centers. A larger pale cell contains multiple dark inclusions marked by asterisks. The image F showing oval cells with dark purple centers. A larger pale cell contains dark inclusions marked by asterisks. A black arrow points from the larger pale cell toward a dense pink clustered mass at the right side.

Navigate back to Figure 5.

## Table 2 Long description

The table is a lower-triangular genetic distance (p-distance) matrix for a 504-bp 18S rDNA fragment comparing 34 Lankesterella sequences identified by GenBank accession and taxon label. Distances among four Lankesterella desseri sequences (OR425022, OR425019, OR425032, OR425028) are extremely small (0.2–0.6%), indicating very close similarity. Another L. desseri cluster (OR425025, OR425018, OR425027) is identical (0.0% between OR425018 and OR425027, and also 0.0% between OR425018 and OR425025/OR425027 where shown), with OR425031 differing by only 0.2% from that cluster. Distances between L. desseri and multiple “Lankesterella sp.” accessions are generally much larger, commonly around 6–11% (for example, MF167553 vs OR425016 is 11.0%, and MF167553 vs OR425025 is 11.2%). The newly labeled Lankesterella nucleoflexa sp. nov. (PX404938) is closest to the OR425021/OR425026/OR425023 L. desseri subgroup at 2.7–2.9%, but is farther from many other L. desseri sequences (about 3.4–4.3% to several OR42501x/OR42502x entries). Several “Lankesterella sp.” sequences are very similar to each other (e.g., MF167544 vs MF167552 is 0.4%; MF167552 vs MF167555 is 0.6%), suggesting additional closely related lineages within the unnamed group. Values are pairwise proportions of differing sites for this single gene fragment, so they indicate relative divergence but do not by themselves establish species boundaries or phylogenetic direction.

Navigate back to Table 2.

## Table 3 Long description

The table compares mean (±SD) and ranges of sporozoite and host-cell measurements (µm) across several haemococcidian taxa, including two iguana parasites from the current study (Lankesterella nucleoflexa nov. sp. and Lankesterella sp.) and multiple previously published species. For intraerythrocytic sporozoites, the current study reports larger lengths (13.60±1.40 and 14.77±0.88) than many earlier values listed (e.g., 6.5–7.3 µm for some species), while L. desseri is similar in size (12.25±1.62). In the current study, infected erythrocytes are larger than uninfected erythrocytes (e.g., area 113.21±14.83 vs 93.27±11.30 for L. nucleoflexa; 90.33±14.44 vs 98±15.91 for Lankesterella sp., noting overlap), and erythrocyte nuclear dimensions are also provided for infected and uninfected cells. Intraleukocytic (monocyte) sporozoites in the current study are notably long (19.13±2.48 for L. nucleoflexa; 16.93±1.89 for Lankesterella sp.) and occur alongside measurements for infected and uninfected monocytes, with infected monocytes showing larger mean area than uninfected in both taxa (131.17±24.92 vs 95.28±39.89; 96.31±31.99 vs 86.33±14.56). Intraleukocytic (heterophil) sporozoites differ strongly between the two iguana taxa (very small in L. nucleoflexa, length 4.54±1.21, versus much larger in Lankesterella sp., 16.58±4.14), and corresponding heterophil size metrics are listed for infected and uninfected cells. Free sporozoites are reported for Lankesterella sp. (length 18.47±2.28; area 47.74±12.73) and are larger than earlier free-sporozoite lengths shown for another taxon (8.6±1.6) and a single reported value (13.0). Many literature columns contain blanks for certain characteristics, so cross-species comparisons are limited to the measurements actually reported, and sample sizes vary by row.

Navigate back to Table 3.
